# The Concentration of Omega-3 Fatty Acids in Human Milk Is Related to Their Habitual but Not Current Intake

**DOI:** 10.3390/nu11071585

**Published:** 2019-07-12

**Authors:** Agnieszka Bzikowska-Jura, Aneta Czerwonogrodzka-Senczyna, Edyta Jasińska-Melon, Hanna Mojska, Gabriela Olędzka, Aleksandra Wesołowska, Dorota Szostak-Węgierek

**Affiliations:** 1 Department of Clinical Dietetics, Faculty of Health Sciences, Medical University of Warsaw, E Ciolka Str. 27, 01-445 Warsaw, Poland; 2 Department of Metabolomics Food and Nutrition Institute, 61/63 Powsińska Str., 02-903 Warsaw, Poland; 3 Department of Medical Biology, Faculty of Health Sciences, Medical University of Warsaw, Litewska Str. 14/16, 00-575 Warsaw, Poland; 4 Laboratory of Human Milk and Lactation Research at Regional Human Milk Bank in Holy Family Hospital, Faculty of Health Sciences, Department of Neonatology, Medical University of Warsaw, Zwirki i Wigury Str. 63A, 02-091 Warsaw, Poland

**Keywords:** human milk, omega-3 fatty acids, docosahexaenoic acid, eicosapentaenoic acid, α-linolenic acid, dietary intake, food frequency questionnaire

## Abstract

This study determined fatty acid (FA) concentrations in maternal milk and investigated the association between omega-3 fatty acid levels and their maternal current dietary intake (based on three-day dietary records) and habitual dietary intake (based on intake frequency of food products). Tested material comprised 32 samples of human milk, coming from exclusively breastfeeding women during their first month of lactation. Milk fatty acids were analyzed as fatty acid methyl ester (FAME) by gas chromatography using a Hewlett-Packard 6890 gas chromatograph with MS detector 5972A. We did not observe any correlation between current dietary intake of omega-3 FAs and their concentrations in human milk. However, we observed that the habitual intake of fatty fish affected omega-3 FA concentrations in human milk. Kendall’s rank correlation coefficients were 0.25 (*p* = 0.049) for DHA, 0.27 (*p* = 0.03) for EPA, and 0.28 (*p* = 0.02) for ALA. Beef consumption was negatively correlated with DHA concentrations in human milk (r = −0.25; *p* = 0.046). These findings suggest that current omega-3 FA intake does not translate directly into their concentration in human milk. On the contrary, their habitual intake seems to markedly influence their milk concentration.

## 1. Introduction

Human milk is universally recognized as the optimal food for infants. Many studies have shown the role of fat in human milk as the main source of energy, selected fatty acids (FAs), crucial fat-soluble vitamins, and key nutrients for the infant development [1,2,3]. Among FAs, polyunsaturated fatty acids (PUFAs) are of principal importance. The two major classes of PUFAs are those of omega-3 and omega-6 families. Omega-3 fatty acids have a carbon–carbon double bond located in the third position from the methyl end of the chain. There are several different omega-3 FAs, but the majority of human milk research focuses on three: docosahexaenoic acid (DHA), α-linolenic acid (ALA), and eicosapentaenoic acid (EPA). ALA contains 18 carbon atoms, whereas EPA and DHA are considered “long-chain” (LC) omega-3 FAs, because EPA contains 20 carbons and DHA contains 22 [4]. Omega-3 FAs, mainly DHA, are important components of retinal photoreceptors and brain cell membranes. Therefore, DHA is essential for infant visual and cognitive development [5,6,7]. The European Food Safety Authority (EFSA) recommends 100 mg/day as the adequate intake of DHA for infants [8].

Fatty acids in human milk may originate either from the maternal dietary FAs, from FAs released from maternal adipose tissue, or from *de novo* synthesis in maternal tissues [5]. The human fatty acid desaturase can form only carbon–carbon double bonds located in the ninth position from the methyl end of a fatty acid. ALA may be endogenously converted to EPA and then to DHA. Therefore, ALA is considered an essential fatty acid, which means that it must be obtained from the diet [9,10]. However, the results of the studies show that the ability to convert ALA to DHA in humans is low, as less than 10% of ALA is converted to DHA [11]. For that reason, DHA from the maternal diet is a much more efficient source of DHA for neural tissue than an equivalent amount of ALA [10,12]. Therefore, consuming EPA and DHA directly from food and/or supplements is the only practical way to increase the levels of these fatty acids in the body.

Many national health authorities [13,14], including the Polish Society for Pediatric Gastroenterology, Hepatology, and Nutrition [15] recommend that maternal intake of DHA should be at least 200 mg per day. Women can meet the recommendation by consuming one to two portions of fatty fish (e.g., salmon, sardines) per week (equivalent of 150–300 g). Although the maternal intake of DHA is crucial for infant brain and retina development, studies carried out in the United States [16], Canada [17,18], and Europe [19] have reported that breastfeeding women do not meet dietary recommendations. This probably results from low fatty fish consumption [20], and is partly related to concerns of methylmercury fish contamination [21], as well as low DHA supplements use [16,17,18].

The tissue levels of FAs in a woman during lactation are directly related to her reserve capacity and the metabolic utilization of fatty acids (synthesis, oxidation and transport). Hence, the maternal diet and metabolism of FAs of women during lactation seem to be the most important factors affecting DHA concentration in human milk. Human milk FA composition changes continuously as dietary FAs are rapidly transported from chylomicrons into human milk with a peak between six and 12 h after dietary DHA intake [21,22].

To investigate the relationship between maternal diet and human milk composition, several dietary assessment methods have been developed and evaluated. The most common are food frequency questionnaires (FFQs) and multiple-day food records. Since food records do not rely on memory, they have been used as a reference method to validate other dietary assessment methods. On the other hand, day-to-day variations and seasonal variations in food consumption may decrease their objectivity. Furthermore, individuals are not always able to recall all the foods consumed or the specific components of the food (especially when dining out), and have difficulty in determining accurate portion sizes and typically underreport dietary intake. This is in contrast to FFQs, which often overestimate the intake of energy and nutrients [23]. Nonetheless, the FFQ has been suggested as an optimal tool in estimating dietary intake of omega-3 fatty acids as it evaluates long-term diet rather than food records [24]. Most of the FFQs available in the literature were designed to assess a wide range of nutrients; however, they were not appropriate for dietary assessment focusing specifically on fatty acids. Given that omega-3 FAs are contained in a particular range of foods, we used a tailored omega-3 FA FFQ. Serra-Majem et al. [25] suggested that its validity is comparable to the whole diet-based FFQs. (0.42–0.52 versus 0.19–0.54). We hypothesize that the concentration of omega-3 fatty acids in human milk is related to their habitual but not current intake; for this reason, in this study, we aimed to determine FA concentrations in maternal milk and assess the association between omega-3 fatty acids levels and their maternal dietary intake evaluated with two methods: dietary intake based on the three-day dietary record, and intake frequency of food products (FFQ, or food frequency questionnaire).

## 2. Materials and Methods 

### 2.1. Subjects and Study Session Design

The Ethics Committee of the Medical University of Warsaw (KB/172/115) approved the study protocol, and all the participating women signed informed consents. A convenience sample of exclusively breastfeeding women (n = 32) was recruited from the Holy Family Hospital in Warsaw. Participants were enrolled during their first month of lactation (weeks two to four). Inclusion criteria comprised: age ≥18 years, singleton pregnancy, and full-term delivery (gestational age ≥37 weeks). Exclusion criteria were as follows: pre-existing chronic or gestational diseases, smoking during pregnancy and/or breastfeeding, low birth weight of the newborn, and low milk supply. The survey consisted of two parts. Firstly, we collected data about socio-demographic and other maternal characteristics, such as: age, education level, material status, pre-pregnancy anthropometric parameters (weight and height), and total weight gain during pregnancy. Then, we collected dietary information involving three-day dietary record and intake frequency of food products. During the study session, the actual body weight and height of every mother were measured using a Seca 799 measurement station and column scales (±0.1 kg/cm; Seca, Chino, CA, USA). The pre-pregnancy and actual body mass index (BMI) was calculated as the ratio of the body weight to the height squared (kg/m^2^). Interpretation of these results followed the international classification proposed by the World Health Organization (WHO): below 18.5 kg/m^2^, underweight; 18.5–24.9 kg/m^2^, normal weight; 25.0–29.9 kg/m^2^, overweight; 30.0 kg/m^2^ and above, obese [26].

### 2.2. Human Milk Collection

Twenty-four-hour human milk samples (n = 32) were collected by women at home after they had been given detailed instructions on taking, storing, and transporting samples to the Holy Family Hospital in Warsaw. Foremilk and hindmilk samples were collected from all the participants from four time periods (06:00–12:00, 12:00–18:00, 18:00–24:00, and 24:00–06:00) to minimize possible circadian influences on the milk fatty acid composition. The term foremilk refers to the milk at the beginning of a feeding, and hindmilk refers to milk at the end of a feeding, which has a higher fat content than the milk at the beginning of that particular feeding. A total of 5 to 10 mL of foremilk and hindmilk samples were obtained from the breast(s) from which the infant was fed. Samples were collected into pre-labeled polypropylene containers provided to each woman. Participants were instructed to store milk in the refrigerator (~4 °C) during the 24-h collection process. Then, milk samples were stored at −20 °C for later analysis.

### 2.3. Lipid Concentration and Fatty Acid Analysis of Human Milk

Tested material comprised 32 samples of human milk. The lipid concentration in human milk was analyzed using the Miris human milk analyzer (HMA) (Miris, Uppsala, Sweden) with a validated protocol, as discussed in a previous study [27]. Collected milk samples for fatty acids analysis were immediately frozen in plastic test tubes at a temperature of −20 °C and delivered to the Department of Metabolomics (Food and Nutrition Institute, Warsaw, Poland) into thermic bags. Samples were stored at −80 °C until analysis. Frozen samples were thawed only once at room temperature, without light. After thawing, samples were shaken at room temperature (3–5 min) to obtain a homogeneous mixture. Aliquots were extracted and analyzed for fatty acid (FA) composition and content.

#### 2.3.1. Fat Extraction

Milk samples were extracted from 1 mL of sample with chloroform-methanol (2:1) (Avantor Performance Materials S.A., Warsaw, Poland) containing 0.02% butyl-hydroxytoluene (2,6-tert-butyl-4-methylphenol, BHT, ≥99.0%, GC, powder) (Sigma-Aldrich CHEMIE GmbH, CA, USA) as an antioxidant, according to Folch et al. [28].

#### 2.3.2. Gas Chromatography-Mass Spectrometry Analysis

Milk fatty acids were analyzed as fatty acid methyl ester (FAME) by gas chromatography using a Hewlett-Packard 6890 gas chromatograph with MS detector 5972 A. The methylation procedure was as follows: organic extracts were evaporated at 40 °C in a gentle nitrogen stream, and then were saponified with 0.5 mL of potassium hydroxide in methanol (0.5 N) (Avantor Performance Materials S.A., Poland) for 10 min at 80 °C in an electric multiblock heater and subsequently methylated with 1 mL of hydrochloric acid in methanol (3 N) (Sigma-Aldrich CHEMIE GmbH, USA) for 15 min at 85 ± 2 °C. After cooling to room temperature, fatty acid methyl esters were extracted with 1 mL of isooctane (2,2,4 trimethylopentane) (Avantor Performance Materials S.A., Poland). One microliter of the sample was injected into the GC column. The GC-MS analysis has been used with a split injector (1:100 ratio), injector and detector temperatures of −250 °C, and carrier gas helium (20 mL/s; the pressure of 43.4 psi). The chromatography oven was programmed to 175 °C for 40 min; thereafter, it was increased by 5 °C per min until the temperature reached 220 °C, and was held at this temperature for 16 min. FAMEs separations were performed on a CP Sil 88 fused silica capillary column (100 m × 0.25 mm i.d., film thickness: 0.20 μm; Agilent J & W GC Columns, CA, USA). Peak identification was verified by comparison with authentic standards (Supelco FAME Mix 37 Component; Sigma-Aldrich, CA, USA) and by mass spectrometry. The obtained results were expressed as a percentage by weight (% *wt/wt*) of all the fatty acids detected with a chain length between eight and 24 carbon atoms. The method was validated and accredited by the Polish Centre of Accreditation (accreditation certificate AB 690). Quality control was also implemented by the use of certified reference materials: BCR-163 (Beef-Pork FAT blend; ABP cat. 3; 8 g; Sigma-Aldrich, CA, USA).

### 2.4. Fatty Acids Dietary Intake and Intake Frequency of Food Products

The assessment of women’s fatty acids intake was based on a three-day dietary record. Mothers were asked to note each food and dietary supplement they had consumed in the tree consecutive days prior to the human milk sampling day. No dietary recommendation was given before the study; participants were allowed to consume self-chosen diets. To verify the sizes of declared food portions, we used the “Album of Photographs of Food Products and Dishes” developed by the National Food and Nutrition Institute [29]. Fatty acids dietary intake was calculated using Dieta 5.0 nutritional software (National Food and Nutrition Institute, Warsaw, Poland). Additionally, the habitual intake of fatty acids was assessed using a FFQ containing 19 items. The FFQ provided information about the consumption frequency of DHA sources in the last three months, such as fish, seafood, meat (poultry, turkey, pork, beef), and eggs. We also collected information about the consumption frequency of other fatty acids sources, including vegetable oils (e.g., canola oil, olive oil, linseed oil, coconut oil), butter, milk and dairy products, nuts, and seeds. The response options were arranged in five categories, from “never”, “less than once a week”, “once or twice a week”, “more than twice a week but not every day”, to “every day”.

### 2.5. Statistical Analysis 

Statistical analyses were performed using Statistica 12PL, Tulusa, USA and IBM Statistics 21, New York, NY, USA. A *p*-value below 0.05 was adopted as statistically significant. Variables distributions were evaluated with a Shapiro–Wilk test and descriptive statistics. Data were presented as means and standard deviations as well as medians and interquartile ranges. Correlations between the intake of fatty acids and fatty acids concentrations in human milk were estimated with Pearson’s r correlation coefficient. Correlations between omega-3 fatty acids (DHA, EPA, ALA) concentrations in human milk, and food consumption frequency were estimated with Kendall’s tau correlation coefficients.

## 3. Results

### 3.1. Maternal Characteristics

The mean maternal age was 30.9 ± 6.5 years and most of them were primiparous (75%; n = 24). Detailed anthropometric data are shown in Table 1. Before pregnancy and during the first month postpartum, none of the participants was classified as being underweight (BMI <18.5 kg/m^2^). In both periods of time, most of them (n = 23, 72%) had normal body mass, and 28% (n = 9) were classified as being overweight or obese. All the participants declared high university education and high material status. We do not observed statistically significant differences between pre-pregnancy and postpartum BMI values (*t*-test was 1.13; *p* = 0.26).

### 3.2. Fatty Acids Concentrations in Human Milk

The fatty acids profile of human milk is shown in Table 2. The relative proportion of saturated, monounsaturated, and polyunsaturated fatty acids was 41.9 ± 4.9%, 39.6 ± 3.1%, and 15.1 ± 3.4%, respectively. The predominant fatty acids in human milk were oleic acid (35.4 ± 3.1%), palmitic acid (19.7 ± 2.5%), and linoleic acid (11.1 ± 2.6%). No significant correlation was found between DHA concentrations, palmitic (r = −0.24; *p* = 0.2) and oleic (r = 0.13; *p* = 0.48) FAs; however, we found correlation with linoleic acid (r = 0.44; *p* = 0.013). Also, a significant negative correlation was found between DHA concentration and the omega-6:omega-3 ratio in human milk (r = −0.45; *p* = 0.012). When only the milk of mothers supplementing their diet with DHA were considered (n = 22), the mean concentration of DHA was 0.78% of total fatty acids. The difference between the concentration of DHA in supplementing and not supplementing mothers was not statistically significant (r = 0.29; *p* = 0.37).

### 3.3. Fatty Acids Dietary Intake and Its Association with Concentration in Human Milk

The fatty acids dietary intake is shown in Table 3. Mean energy intake was 1752 kcal ± 228.3 kcal, which was lower than the recommended level (EER, estimated energy requirement = 2555 kcal per day). The risk of deficient energy intake was observed in 100% of the participants. According to the Polish nutritional standards, the recommended intake for ALA is 0.5% of total energy, which in our participants it corresponded to 0.97 g per day. The mean dietary intake of ALA (1.5 ± 0.8% of total fatty acids) was higher than recommended levels; nevertheless, 22% of participants did not meet the recommendation. When only dietary sources were considered, the mean intake of DHA (243 mg ± 333.5) (Table 4) reached the Polish [15] and European [13,14] recommendation of 200 mg of DHA daily, whereas among 59% of the women, we observed insufficient DHA intake. Including taken supplements, the percentage of deficient DHA intake decreased, and was 16%. The majority of the participants (69%; n = 22) reported taking DHA supplements, and 10% (n = 3) of women declared taking supplements containing DHA and EPA.

Table 5 presents correlation coefficients (Pearson’s r) between human milk fatty acids concentrations and maternal fatty acids dietary intake, as well as maternal dietary intake together with supplementation. We did not observe any statistically significant correlation between these factors (*p* > 0.05).

### 3.4. Association between Intake Frequency of Food Products and DHA Concentrations in Human Milk

Table 6 presents Kendall’s rank correlation coefficients between the intake frequency of food products and DHA concentrations in human milk. According to the FFQ, almost half of the participants (~47%) declared fatty fish consumption once or twice a week. On the other hand, almost 43% of the women consumed fish less than once a week or never. Based on the three-day dietary record, the most frequently consumed fatty fish species were salmon and mackerel, which were reported by seven (22%) and six women (19%), respectively. Butter and milk were the most frequently used foods, which were consumed by approximately ~44% and ~38% of participants, respectively.

We found a significant positive correlation between fatty fish consumption and all the omega-3 fatty acids concentrations in human milk. Kendall’s rank correlation coefficients were 0.25 (*p* = 0.049) for DHA, 0.27 (*p* = 0.03) for EPA, and 0.28 (*p* = 0.02) for ALA. The ALA content in maternal milk was also positively correlated with the intake frequency of linseed oil (r = 0.30; *p* = 0.01), coconut oil (r = 0.29; *p* = 0.02), milk (r = 0.26; *p* = 0.04), and fermented dairy products (r = 0.29; *p* = 0.02), whereas EPA concentration was positively correlated with intake frequency of pork (r = 0.29; *p* = 0.02). Beef consumption, by contrast, was negatively correlated with DHA concentration in human milk (r = −0.25; *p* = 0.046). No other significant correlations between intake frequency of food products and DHA concentration in human milk were found.

**Figure 1:**
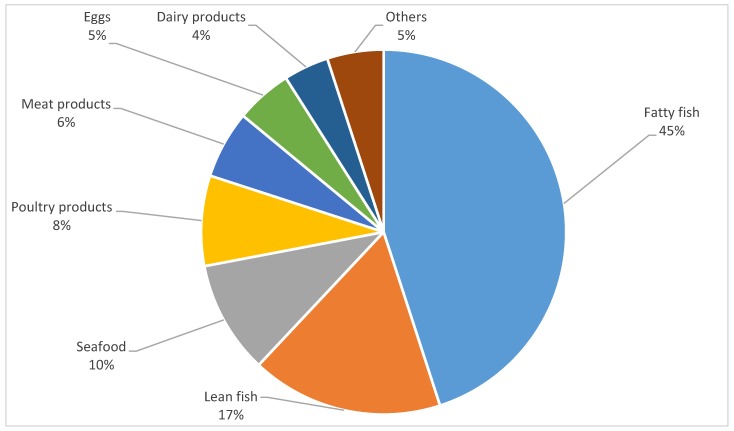
The relative contribution of food groups to total omega-3 FAs intake from the three-day dietary record.

The major food contributor of total omega-3 FAs intake was fatty fish (45%) and lean fish (17%) (Figure 1). Seafood (mainly shrimps) (10%), poultry products (8%), and meat products (6%) also made significant contributions to the estimated intake of omega-3 FAs. Within seafood and fish categories, salmon was found to be the main source of all omega-3 FAs, DHA (67%), EPA (53%), and ALA (59%).

Food items were divided into groups based on the Dieta 5.0 nutritional software database. Food items that contributed to less than 1% of total omega-3 FAs intake were categorized as “others”. This included fats and vegetable oils, mixed dishes, and sauces.

## 4. Discussion

Our study has revealed three primary findings concerning the studied population. First, the mother’s dietary intake of omega-3 FAs met Polish and European standards. Secondly, milk DHA concentration averaged about 0.70% of total fatty acids, which was twofold higher than the worldwide average (WWA) [30]. The third finding of this study is that there were no correlations between dietary intake of omega-3 FAs measured with three-day dietary records and their concentrations in human milk, whereas the intake frequency of food products—mainly fatty fish—was positively correlated with the concentration of ALA, EPA, and DHA in human milk.

In our study, the average maternal dietary DHA and ALA intakes (including taken supplements) were 613 ± 575 mg and 152 ± 0.81 mg, respectively. For DHA, this value was threefold higher than Polish [15] and European [13,14] recommendations (200 mg DHA per day). Our finding is not consistent with previous studies conducted in the United States [31,32], Canada [17,33], and Europe [34], which reported that the majority of breastfeeding women in the Alberta Pregnancy Outcomes and Nutrition (APrON) cohort were not meeting any of the various authorities’ omega-3 FAs recommendations [35]. Similarly, in the Latvian study (Latvia and Poland are situated in the same geographic region, both countries have access to the Baltic Sea), DHA daily intake was lower than recommended, and was only 136 ± 26 mg [36]. It is widely reported that supplementation increases mother’s milk DHA concentrations [37,38], and our results confirmed these findings. DHA concentration in the milk of mothers who supplemented their diet with DHA was higher than in the whole group, and was 0.78% of total fatty acids. However, we did not observe statistically significant differences between DHA concentrations in the milk of mothers who supplemented and did not supplement their diet with DHA (r = 0.29; *p* = 0.37). When only dietary sources were considered, the insufficient intake of DHA was observed among 59% of participants, whereas including taken supplements, the percentage decreased to 16%.

Based on the three-day dietary record, we observed that the largest contributor of omega-3 FAs intake was fatty fish, lean fish, and seafood, which was consumed once or twice a week by 47%, 44%, and 25% of participants, respectively. Other foods significantly contributing to total omega-3 FAs intake were poultry, red meat (pork and beef), and eggs. These findings were consistent with other studies [18,19], which reported that seafood, fish, poultry, meat, and eggs were the primary dietary sources of total omega-3 FAs.

The mean concentration of DHA in human milk in our study (0.7 ± 0.3% of total fatty acids) was higher than those reported in the meta-analysis based on 106 studies published between 1986–2006 year [30]. The authors found that the WWA DHA concentration in human milk was 0.32 ± 0.22% with a wide range of 0.06% to 1.4% of total fatty acids. We observed that our result was twofold compared to the data from Mediterranean countries, such as Italy (0.28–0.35%) [39,40], Spain (0.31–0.38) [41,42], and also Nordic countries such as Iceland (0.30%) [43] and Denmark (0.35%) [44]. Our mean DHA concentration in human milk was also higher than that reported by Jackson and Harris [45]. They suggested that the target of DHA level in human milk should be 0.3% of total fatty acids, emphasizing however that the optimal DHA level remains to be established. An approximate DHA concentration in human milk may be achieved when DHA intake exceeds 200 mg. Brenna and Lapillonne [14] have developed a regression analysis equation to calculate human milk DHA concentration depending on dietary DHA intake, based on the data from Gibbson et al. [46]:

human milk DHA concentration (% of total fatty acids) = 0.72 × maternal DHA intake (g per day) + 0.20.

Inserting our mean maternal DHA intake into this equation, we obtain a value of 0.63%, which is similar to the mean DHA concentration in human milk from our analysis (0.7%). In our study, the daily DHA intake was significantly higher than recommended, which may explain that the target DHA level in human milk was reached.

In our study, the mean concentration of ALA in human milk was 1.5 ± 0.8%, which was higher than those reported by Presa-Owens et al. (0.79%) [47] and Kotelzko et al. (0.81%) [48]. Some oils, such as soybean oil and canola oil, and legumes such as soybeans contain large amounts of ALA. Our findings suggest that women in our study consume these types of products to a greater extent than those in other Western countries [49]. However, in our study, the ratio of LA to ALA in maternal milk was higher than recommended (2.8:1) [50], and was 8:1. As LA and ALA compete for the same key enzymes in long-chain polyunsaturated fatty acids (LCPUFAs) biosynthesis, and ALA has a higher affinity than LA for ð-6-desaturase, the dietary ratio of LA:ALA is more relevant for proper LCPUFAs production than the intake of each fatty acid individually [49]. Furthermore, there is evidence that the DHA concentration in maternal milk has a positive correlation with the intelligence quotient of the child, whereas LA concentration has a negative correlation. It indicates that the FA profile during the lactation period is of particular significance [51,52,53]. In our study, the ratio of DHA:LA (0.1:1) was higher than the mean ratio from 28 countries [54]. Lassek and Gaulin [54] reported that the DHA:LA ratio at the level of ~0.04:1–0.07:1 is linked to higher cognitive test score results. It is worth noting that in our study, the majority of women (94%, n = 30) had the DHA:LA ratio in their milk of at least 0.04:1. Further, their mean ratio of omega-6:omega-3 FAs (4.6:1) was three times lower than the value reported by Silva et al. (14:1) [55], and nearly two times lower than that in the Latvian study (7:1) [36]. Silva et al. [52] and Nishimura et al. [56] suggested that a diet low in fish and high in vegetable oils, mainly soybean oil, facilitated a higher omega-6:omega-3 ratio (>10:1). However, we did not observe a significant correlation between fish or vegetable oils consumption and the omega-6:omega-3 ratio in human milk. The EFSA recommended a 4:1 proportion for dietary omega-6:omega-3 FAs [8]. It has been suggested that a very high omega-6:omega-3 ratio (~15:1) may promote cardiovascular diseases and cancers [57].

We found a significant positive correlation (r = 0.25; *p* = 0.049) between habitual fatty fish consumption and DHA concentration in human milk, which is consistent with results from other studies [36,49,56,58]. A Danish observational study reported a higher proportion of DHA concentration in the milk of mothers who consumed fish than in those who did not consume fish (0.63% compared with 0.41%; *p* = 0.018) and in women who consumed fatty fish compared with those who did not consume fish (0.73% compared with 0.41%; *p* < 0.01) [59]. Contrary to these findings, Juber et al. [59] reported that intake of fish did not correlate with mother’s milk DHA levels. The results may depend on fish breeding (farmed or wild) and the fish species that are most commonly consumed in a specific region. For instance, when consuming 100 g of wild salmon, the total DHA intake is lower (0.31% of total fatty acids) compared to eating the same quantity of farmed salmon (0.88% of total fatty acids). It is caused by the higher total fat content in farmed salmon (12.3 g/100 g) than in wild salmon (2.07 g/100 g) [60]. In our study, the most commonly consumed fish were salmon and mackerel, but we did not have information about breeding, so the dietary intake of DHA based on three-day dietary record may be overestimated on underestimated in some cases. Using Dieta 5.0 nutritional software, we based our analysis on its nutritional database, which did not have distinctions between wild and farmed fish. However, Qiunn and Kuawa [61] concluded that an additional portion of fish (no information about breeding) per week led to a 0.014% increase of DHA concentration in human milk. We also observed that the DHA levels in human milk were negatively correlated (r = −0.25; *p* = 0.046) with beef consumption, the fat of which consists mainly of saturated fatty acids (SFA) (~52.3 g/100 g) and contains low amounts of PUFA (3.8 g/100 g) [62].

We noted that similarly to DHA, the ALA and EPA concentrations in human milk were also significantly positively correlated with the frequency of fatty fish consumption (r = 0.27; *p* = 0.02 for ALA and r = 0.28; *p* = 0.03 for EPA). ALA concentration in human milk was also positively correlated with the frequency of consuming linseed oil, coconut oil, milk, and milk products. Milk fat consists mainly of SFA (59.4 g/100 g) with the dominating palmitic acid (~30.6% of total FAs) [63], which was predominant SFA in human milk in our study. However, no significant correlation between milk consumption and palmitic acid concentration in human milk was found (r = 0.13; *p* = 0.29).

We did not find a correlation between dietary intake of DHA based on the three-day dietary record and any omega-3 FAs (ALA, EPA, and DHA) concentrations in human milk. It confirmed the results of Nishimura et al. [55], who did not observe any association between dietary EPA and DHA intake during the postpartum period and their concentrations in human milk. However, they noted that the dietary EPA and DHA content during the third trimester of pregnancy was directly related to the content of these fatty acids in mature human milk. These results suggest that maternal body stocks of fatty acids have a greater influence on breast milk fatty acid composition than the estimated dietary intake during the postpartum period, validating previously reported results [2,64]. An additional possible explanation would be also the increased weight gain and body fat storage observed in women mainly during late pregnancy [65]. Fiddler et al. [66] reported that about 20% of the supplemented 0.2 g of DHA per day was secreted into milk and observed a pronounced increase in DHA content in human milk (% of total FAs), with a peak between 6–12 h after dietary DHA intake. Although 24-h human milk samples were collected in our study, these findings may explain the lack of correlation between the intake of omega-3 FAs measured with the three-day dietary record and its concentration in human milk. Further, while the synthesis of DHA from ALA may be sufficient for the healthy breastfeeding women, some non-physiological conditions, such as non-alcoholic fatty liver, which reduces the activity of ð-6-desaturase enzymes, could contrary affect the synthesis of DHA, decreasing the levels of omega-3 FAs in erythrocytes and then in human milk [67]. Moreover, the endogenous synthesis of PUFAs is mediated by genes controlling the elongation of very long chain fatty acids protein 2 and 5 (ELOVL2 and ELOVL5) [68]. It is also suggested that the genes encoding fatty acids desaturases 1 and 2 (FADS1 FADS2 gene cluster) were reported to be associated with omega-3 and omega-6 FA proportions in human plasma, tissues, and milk [69]. Moltó-Puigmartí et al. [69] found that DHA proportions were lower in women homozygous for the minor allele than in women who were homozygous for the major allele (DHA proportions in plasma phospholipids: *p* < 0.01; DHA proportions in milk: *p* < 0.05). Contrary to our and previous citied studies, a Greek observational study reported that human milk omega-3 PUFA content was positively correlated with maternal dietary intakes of total PUFAs (r = 0.26; *p* < 0.05), inversely correlated with maternal carbohydrates intake (r = −0.29; *p* < 0.05), and unrelated to maternal energy, total fat, SFA, MUFA, and protein intakes [37]. Bravi et al. [70] carried out a systemic review searching studies investigating the impact of maternal diet on their milk composition. Considering fatty acid profiles, most of the studies were based on supplements intervention or assessed maternal nutritional status with a food frequency questionnaire. As the authors concluded, the gathered results were scarce and diversified.

The time of incorporation of omega-3 FAs into plasma lipids is quite long, and similar to their half-life. The longest time of incorporation is in adipose tissue, which is an important source of plasma lipids. As the content of omega-3 FAs in human milk depends on its availability from the plasma [71], dietary intake assessment methods studying habitual intake are more appropriate to exhibit the discussed correlation. Thus, the survey based solely on a three-day dietary record, especially in this case, does not work. This is not only because of the described omega-3 FAs kinetics, but also because omega-3 FAs food sources (mainly fish) are not consumed every day, and whether they happen to occur in the three-day period is a matter of chance.

The strengths of this study are the use of milk collection protocol, which enabled minimizing possible errors in the measurement of human milk composition, and also an assessment of maternal diet with two techniques (food frequency questionnaire and three-day dietary record). Additionally, contrary to other authors [56], we assayed α-linolenic acid that can convert to DHA; therefore, a correlation between dietary intake of DHA and its concentration in human milk is more reliable. Our study has also limitations. First, there were the issues of convenience sampling and the modest number of participants, resulting from the complex nutrition questionnaire and milk collection protocol. Second, this study was conducted in a large city (the capital of Poland); all the participants had a university education and high material status. Third, genetic polymorphisms that influence the use of PUFAs from the diet have not been studied. These factors may limit the generalization of our findings to a broader population, because the availability and consumption patterns of food products (e.g., fish and seafood) is strongly related to the place of living and material status.

## 5. Conclusions

To summarize, this study shows that the women under study during breastfeeding had an adequate intake of foods that are natural sources of omega-3 FAs (fatty fish, seafood, vegetable oils), which resulted in a high concentration of DHA in their milk. However, it should be stressed that we did not observe a correlation between dietary intake of omega-3 FAs and their concentrations in human milk. On the other hand, we observed that the intake frequency of some food products affected omega-3 FAs concentrations in human milk. Considering these findings and the highest content of DHA in human milk being observed between 6–12 h after dietary DHA intake, a short-term assessment of omega-3 FAs intake (based on three-day dietary record) may be unreliable. A FFQ assessing nutritional habits in the last three months prior to the study seems to the more reliable tool, reflecting the habitual intake of omega-3 FAs.

## Figures and Tables

**Table 1:** Characteristics of the mothers.

Characteristic	Mean ± SD	Range
Age (years)	30.9 ± 6.5	27–44
Height (cm)	1.66 ± 0.1	1.54–1.8
Pre-pregnancy weight (kg)	62.2 ± 11.8	44–90
Pre-pregnancy body mass index (kg/m^2^)	22.6 ± 3.4	18.6–30.9
Weight gain during pregnancy (kg)	15.1 ± 4.8	7–30
Weight at first month postpartum (kg)	65.5 ± 13.2	45.6–95
Body mass index at first month postpartum (kg/m^2^)	23.6 ± 3.8	18.5–32.1

**Table 2:** Fatty acids composition (%) of human milk ^1^.

Fatty Acids	Mean ± SD	Median (Interquartile Range)
**Saturated fatty acids (SFA)**	41.9 ± 4.9	42.3 (38.0–45.7)
C4:0 (butanoic acid)	0.0 ± 0.0	0.0 (0.0–0.0)
C6:0 (caproic acid)	0.0 ± 0.0	0.0 (0.0–0.0)
C8:0 (caprylic acid)	0.1 ± 0.0	0.1 (0.1–0.1)
C10:0 (capric acid)	1.1 ± 0.3	1.1 (1.0–1.4)
C12:0 (lauric acid)	3.5 ± 1.1	3.3 (2.5–4.1)
C13:0 (tridecanoic acid)	0.1 ± 0.0	0.1 (0.0–0.1)
C14:0 (myristic acid)	9.5 ± 2.5	9.3 (7.9–11.1)
C15:0 (pentadecanoic acid)	0.8 ± 0.2	0.7 (0.7–0.8)
C16:0 (palmitic acid)	19.7 ± 2.5	19.9 (17.4–22.0)
C17:0 (margaric acid)	0.5 ± 0.1	0.5 (0.4–0.6)
C18:0 (stearic acid)	6.4 ± 1.5	6.0 (5.4–7.3)
C20:0 (arachidic acid)	0.2 ± 0.1	0.2 (0.1–0.2)
C21:0 (henicosanoic acid)	0.0 ± 0.0	0.0 (0.0–0.0)
C22:0 (behenic acid)	0.1 ± 0.0	0.1 (0.1–0.1)
C23:0 (tetracosanoic acid)	0.0 ± 0.0	0.0 (0.0–0.0)
**Monounsaturated fatty acids (MUFA)**	39.6 ± 3.1	39.0 (38.0–42.0)
C14:1 (myristoleic acid)	0.2 ± 0.1	0.2 (0.2–0.3)
C15:1 (pentadecenoic acid)	0.0 ± 0.0	0.0 (0.0–0.0)
C16:1 trans	0.4 ± 0.1	0.4 (0.3–0.4)
C16:1 cis	2.6 ± 0.5	2.6 (2.3–2.9)
C17:1 (heptadecenoic acid)	0.2 ± 0.0	0.2 (0.2–0.3)
C18:1 cis (oleic acid)	35.4 ± 3.1	34.9 (33.6–37.6)
C18:1 trans (vaccenic acid)	1.2 ± 0.5	1.2 (0.8–1.5)
C20:1 (gadoleic acid)	0.8 ± 0.2	0.7 (0.7–0.9)
C22:1 (erucic acid)	0.2 ± 0.1	0.1 (0.1–0.2)
C24:1 (lignoceric acid)	0.2 ± 0.0	0.2 (0.1–0.2)
**Polyunsaturated fatty acids (PUFA)**	15.1 ± 3.4	15.3 (12.7–16.8)
n-3 polyunsaturated	2.7 ± 0.9	2.6 (2.1–3.1)
C18:3 (α-linolenic acid, ALA)	1.5 ± 0.6	1.4 (1.0–1.8)
C20:3 (eicosatrienoic acid)	0.1 ± 0.0	0.1 (0.0–0.1)
C20:5 (eicosapentaenoic acid, EPA)	0.2 ± 0.1	0.2 (0.2–0.3)
C22:6 (docosahexaenoic acid, DHA)	0.7 ± 0.3	0.7 (0.5–1.0)
**n-6 polyunsaturated**	12.1 ± 2.7	12.1 (10.4–13.4)
C18:2 (linoleic acid, LA)	11.1 ± 2.6	11.1 (9.5–12.3)
C18:3 (γ-linoleic acid)	0.1 ± 0.0	0.1 (0.1–0.1)
C20:3 (dihomo-γ-linoleic acid)	0.3 ± 0.1	0.3 (0.2–0.4)
C20:4 (arachidonic acid, ARA)	0.5 ± 0.1	0.5 (0.4–0.6)
**Ratio**		
n-6:n-3	4.6 ± 1.0	4.8 (4.1–5.1)
DHA:LA ^2^	0.1 ± 0.0	0.1 (0.0–0.1)
ARA ^3^:DHA ^4^	0.9 ± 0.4	0.7 (0.5–1.1)
LA:ALA ^5^	8.1 ± 2.4	7.8 (6.4–9.6)
Total fat concentration ^6^	3.49 ± 1.0	3.5 (3.0–4.2)

^1^ Data are presented as the relative proportion of each fatty acid (% of total fatty acids). ^2^ LA linoleic acid; ^3^ ARA arachidonic acid; ^4^ DHA docosahexaenoic acid; ^5^ ALA α-linolenic acid. ^6^ Total fat concentration is presented as grams per 100 mL.

**Table 3:** Fatty acids content in mothers’ diet.

Fatty Acids	Mean ± SD (g)	Median (Interquartile Range) (g)
Saturated fatty acids (SFA)	23.9 ± 10.3	20.9 (7.3–69.5)
C4:0 (butanoic acid)	0.4 ± 0.3	0.5 (0–1.1)
C6:0 (caproic acid)	0.3 ± 0.2	0.3 (0–0.8)
C8:0 (caprylic acid)	0.2 ± 0.2	0.2 (0–1.0)
C10:0 (capric acid)	0.5 ± 0.3	0.5 (0–1.6)
C12:0 (lauric acid)	0.9 ± 0.9	0.7 (0.2–5.5)
C14:0 (myristic acid)	2.8 ± 1.4	2.7 (0.7–7.9)
C15:0 (pentadecanoic acid)	0.3 ± 0.2	0.3 (0–0.9)
C16:0 (palmitic acid)	12.6 ± 5.2	11.6 (4.5–35.0)
C17:0 (margaric acid)	0.2 ± 0.1	0.2 (0–0.7)
C18:0 (stearic acid)	5.2 ± 2.8	5.1 (1.2–18.3)
C20:0 (arachidic acid	0.1 ± 0.1	0.1 (0–0.3)
**Monounsaturated fatty acids (MUFA)**	24.7 ± 8.9	24.8 (13.2–55.0)
C14:1 (myristoleic acid)	0.2 ± 0.2	0.2 (0–0.8)
C15:1 (pentadecenoic acid)	0.1 ± 0.1	0.1 (0–0.2)
C16:1	1.3 ± 0.4	1.2 (0.6–2.4)
C17:1 (heptadecenoic acid)	0.1 ± 0.1	0.1 (0–0.5)
C18:1 (oleic and vaccenic acids)	22.1 ± 8.3	21.7 (11.5–50.3)
C20:1 (gadoleic acid)	0.3 ± 0.2	0.2 (0.1–0.9)
C22:1 (erucic acid)	0.3 ± 0.3	0.1 (0–1.1)
**Polyunsaturated fatty acids (PUFA)**	10.7 ± 4.0 13.5 ± 14.2 ^s^	11.2 (4.9–22.9) 11.3 (4.9–88.4) ^s^
C18:2 (linoleic acid, LA)	8.5 ± 3.4	8.5 (4.1–21.1)
C18:3 (α-linolenic acid, ALA)	1.5 ± 0.8	1.4 (0.5–4.3)
C20:5 (eicosapentaenoic acid, EPA)	0.1 ± 0.2 0.1 ± 0.2 ^s^	0 (0–0.5) 0.1 (0–0.5) ^s^
C22:6 (docosahexaenoic acid, DHA)	0.2 ± 0.3 0.6 ± 0.6 ^s^	0.1 (0–1.2) 0.4 (0–1.8) ^s^

^s^ Diet + supplementation.

**Table 4:** Estimated daily intake of EPA and DHA from food, supplement, and food + supplement in lactating women.

	EPA (mg)	DHA (mg)
Food	104.4 ± 152.0 (18, 0–190)	243.3 ± 333.5 (50, 37–411)
Supplement ^1^	29.7 ± 43.6 (0, 0–43)	370.6 ± 465.1 (250, 8–549)
Food + supplement	134.1 ± 153.9 (85, 1–215)	614.0 ± 574.6 (354, 202–905)

Data are presented as means ± SD (median, interquartile range). ^1^ Participants who did not take supplements or those who took supplements that did not contain EPA/DHA were considered as 0 mg supplement on EPA and DHA.

**Table 5:** Correlations between human milk fatty acids and fatty acids in the mother’s diet and supplementation.

Concentration in Human Milk	Dietary Intake, Sole or Together with Supplementation					
	SFA	MUFA	PUFA	ALA	EPA	DHA
SFA ^1^	0.26	0.16	−0.20	−0.13	−0.18 −0.19 ^s^	−0.10 −0.16 ^s^
MUFA ^2^	−0.14	−0.04	0.14	0.26	0.21	0.19
PUFA ^3^	−0.20	−0.14	0.20	0.01	0.08	−0.01
ALA ^4^	−0.19	−0.09	0.04	0.32	0.20	−0.06
EPA ^5^	−0.16	0.05	0.05	−0.11	0.20 0.17 ^s^	−0.02 0.17 ^s^
DHA ^6^	−0.24	−0.18	−0.04	−0.26	0.16 0.23 ^s^	0.04 0.24 ^s^

Data are presented as Pearson’s r coefficients.; ^1^ SFA, Saturated fatty acids; ^2^ MUFA, Monounsaturated fatty acids; ^3^ PUFA, Polyunsaturated fatty acids; ^4^ ALA, α-linolenic acid; ^5^ EPA, eicosapentaenoic acid; ^6^ DHA, docosahexaenoic acid; ^s^ Pearson’s r coefficients diet + supplementation.

**Table 6:** Intake frequency (%) of food products and correlations with DHA concentrations in human milk ^1^.

Food	Never	Less than Once a Week	Once or Twice a Week	More than Twice a Week but Not Every Day	Every Day	Correlation ^1^ with Concentrations in Human Milk		
						DHA ^2^	EPA ^3^	ALA ^4^
Fatty fish (e.g., salmon, herring)	12.50	31.25	46.88	9.38	0.00	0.25 *	0.27 *	0.28 *
Lean fish (e.g., cod, sole)	21.88	31.25	43.75	3.13	0.00	0.14	0.08	0.21
Seafood	31.25	43.75	25.17	0.00	0.00	0.21	0.13	0.19
Poultry and turkey	3.13	3.13	43.75	40.63	9.38	0.09	0.13	0.08
Pork	6.25	40.63	37.50	15.63	0.00	0.02	0.29 *	0.18
Beef	9.38	50.00	34.38	6.25	0.00	−0.25 *	−0.14	0.11
Meat products (e.g., sausages, sliced meats)	6.25	12.50	37.50	18.75	25.00	0.24	−0.11	−0.10
Eggs	15.63	6.25	34.38	34.38	9.38	−0.14	−0.06	−0.00
Milk	31.25	15.63	9.38	6.25	37.50	0.02	0.05	0.26 *
Fermented dairy products	37.50	28.13	21.88	12.50	0.00	0.17	0.10	0.29 *
Cheese	25.00	28.13	15.63	18.75	12.50	−0.02	−0.02	0.00
Cottage cheese	21.88	12.50	43.75	15.63	6.25	0.22	0.06	0.17
Milk desserts	46.88	28.13	9.38	15.63	0.00	0.11	−0.12	−0.18
Butter	15.63	9.38	12.50	18.75	43.75	−0.14	−0.11	0.10
Canola oil	18.75	28.13	21.88	25.00	6.25	0.09	−0.0	0.13
Olive oil	12.50	18.75	25.00	43.75	0.00	0.08	0.02	0.03
Linseed oil	62.50	15.63	9.38	9.38	3.13	0.01	0.10	0.30 *
Coconut oil	56.25	25.00	15.63	3.13	0.00	−0.12	−0.07	0.29 *
Nuts and seeds	6.25	18.75	21.88	28.13	25.00	−0.03	0.02	0.01

^1^ Data are presented as Kendall’s rank correlation coefficients. * *p* < 0.05. ^2^ DHA, docosahexaenoic acid; ^3^ EPA, eicosapentaenoic acid; ^4^ ALA, α-linolenic acid.
